# Taurine chloramine differentially inhibits matrix metalloproteinase 1 and 13 synthesis in interleukin-1β stimulated fibroblast-like synoviocytes

**DOI:** 10.1186/ar2279

**Published:** 2007-08-14

**Authors:** Kyoung Soo Kim, Eun Kyung Park, Seung Min Ju, Hye-Sook Jung, Jun Soo Bang, Chaekyun Kim, Yeon-Ah Lee, Seung-Jae Hong, Sang-Hoon Lee, Hyung-In Yang, Myung Chul Yoo

**Affiliations:** 1 East-West Bone & Joint Research Center, East-West Neo Medical Center, Kyung Hee University, Sangil-dong, Gangdong-gu, Seoul, Republic of Korea; 2 Center for Advanced Medical Education by BK21 Project, Inha University School of Medicine, Incheon, Republic of Korea; 3 Department of Internal Medicine, College of Medicine, Kyung Hee University, Hoegi-1-dong, Dongdaemun-gu, Seoul, Republic of Korea; 4 Department of Internal Medicine, East-West Neo Medical Center, Kyung Hee University, Sangil-dong, Gangdong-gu, Seoul, Republic of Korea; 5 Department of Orthopedic Surgery, East-West Neo Medical Center, Kyung Hee University, Sangil-dong, Gangdong-gu, Seoul, Republic of Korea

## Abstract

It has been suggested that taurine chloramine (TauCl) plays an important role in the downregulation of proinflammatory mediators. However, little is known about its effect on the expression of matrix metalloproteinases (MMPs). In this study, we investigated the effects of TauCl on synovial expression of MMPs. The effects of TauCl on MMP expression in IL-1β stimulated fibroblast-like synoviocytes (FLSs) were studied using the following techniques. Real-time PCR and semi-quantitative PCR were employed to analyze the mRNA expression of MMPs. ELISA was used to determine protein levels of MMPs. Western blot analyses were performed to analyze the mitogen-activated protein kinase and inhibitor of nuclear factor-κB (IκB) kinase signalling pathways. Finally, electrophoretic mobility shift assay and immunohistochemistry were used to assess localization of transcription factors. IL-1β increased the transcriptional and translational levels of MMP-1 and MMP-13 in rheumatoid arthritis FLSs, whereas the levels of MMP-2 and MMP-9 were unaffected. TauCl at a concentration of 400 to 600 μmol/l greatly inhibited the transcriptional and translational expression of MMP-13, but the expression of MMP-1 was significantly inhibited at 800 μmol/l. At a concentration of 600 μmol/l, TauCl did not significantly inhibit phosphorylation of mitogen-activated protein kinase or IκB degradation in IL-1β stimulated rheumatoid arthritis FLSs. The degradation of IκB was significantly inhibited at a TauCl concentration of 800 μmol/l. The inhibitory effect of TauCl on IκB degradation was confirmed by electrophoretic mobility shift assay and immunochemical staining for localization of nuclear factor-κB. TauCl differentially inhibits the expression of MMP-1 and MMP-13, and inhibits expression of MMP-1 primarily through the inhibition of IκB degradation, whereas it inhibits expression of MMP-13 through signalling pathways other than the IκB pathway.

## Introduction

The characteristics of rheumatoid arthritis (RA) include chronic proliferative synovitis, infiltration of inflammatory immune cell types into the synovial fluid of joints, and cartilage destruction. Proliferative fibroblast-like synoviocytes (FLSs) play crucial roles in both joint damage and propagation of inflammation because they produce many mediators of inflammation and matrix metalloproteinases (MMPs), which contribute to cartilage degradation in joints [1]. Immune cells recruited into joint cavities by FLSs also contribute to progressive destruction of cartilage in distal joints [2]. Among the range of detrimental immune cells that are present in RA joints, neutrophils have been a primary focus of research in RA because of their number and function [3-7]. Once activated, neutrophils secrete various mediators, including MMPs and, in particular, the reactive oxygen intermediates nitric oxide and hypochlorous acid (HOCl) [8,9]. Thus, neutrophils play an important role in the pathogenesis of RA [9].

However, neutrophils also appear to possess homeostatic mechanisms that can reduce the inflammatory response. Activated neutrophils contain substantial quantities of taurine, which is one of the most abundant free intracellular amino acids present in mammalian tissues and blood cells [10,11]. Taurine acts as a scavenger of HOCl, which is produced by the myeloperoxidase/hydrogen peroxide/chloride system of activated neutrophils and monocytes [12]. It reacts with HOCl to form taurine chloramine (TauCl). Notably, TauCl has been shown to play a major role in downregulating the expression of inflammatory mediators such as chemokines, cytokines, cyclo-oxygenase-2 and inducible nitric oxide synthase in various types of cells [13-18]. Such inhibitory effects have also been demonstrated in animal models of arthritis [19,20]. These inhibitory effects may stem from the suppressive effects of TauCl on expression of proinflammatory mediators (prostaglandin E_2_, nitric oxide, and cytokines) and bone erosion related enzymes, such as MMPs.

MMPs, which are primarily produced in fibroblast-like synoviocytes (FLSs) in RA, are proteases that participate in irreparable proteolytic degradation and in the remodelling of the extracellular matrix. MMPs can be classified into five main groups, according to their substrate specificities, primary structures and cellular localizations [21]: collagenases (MMP-1, MMP-8 and MMP-13), gelatinases (MMP-2 and MMP-9), stromelysins (MMP-3 and MMP-10), matrilysins (MMP-7 and MMP-26) and membrane-bound membrane-type MMPs (MMP-14, MMP-15, MMP-16, MMP-17, MMP-24 and MMP-25). The MMP-1 and MMP-13 collagenases play dominant roles in RA and osteoarthritis because they are rate-limiting components of the collagen degradation process [22,23]. In particular, MMP-13 is a potent protease that is capable of degrading a wide range of collagenous and noncollagenous extracellular matrix macromolecules [24,25]. MMP-13 is remarkably active against collagen type II, which is the predominant collagen in cartilage [26]. To date, investigations of TauCl have focused on its inhibitory effects on the expression of proinflammatory mediators. However, despite the important roles played by MMPs in cartilage erosion, the effects of TauCl on expression of MMPs are not well understood. In this report we show that TauCl inhibits the increased expression of the MMP-1 and MMP-13 genes in IL-1β stimulated RA FLSs.

## Materials and methods

### Primary culture of fibroblast-like synoviocytes

After obtaining informed consent, synovial tissues were collected from RA patients who met the 1987 American College of Rheumatology criteria for the diagnosis of RA and who were undergoing therapeutic joint surgery. FLSs were isolated as follows. Tissues were digested with gentle shaking in 20 ml RPMI 1640 (Gibco-BRL, Grand Island, NY, USA) containing 1 mg/ml collagenase (Gibco-BRL) at 37°C for 90 min, filtered through a 70 μm cell strainer and cultured in 75 cm^2 ^culture flasks with Dulbecco's modified essential medium (Gibco-BRL) supplemented with 20% (vol/vol) foetal bovine serum (Gibco-BRL) and 1× antibiotic-antimycotic (Gibco-BRL). After the cells had grown to confluence, they were detached with 0.25% trypsin (Gibco-BRL) and split at a 1:4 ratio. FLS passages three to six were used for all experiments. Visual examination of cell morphology under light microscopy and fluorescence activated cell sorting analysis of cells stained with anti-CD11b antibody (Santa Cruz Biotechnology, Santa Cruz, CA, USA) confirmed that FLSs accounted for more than 95% of the cells.

### Preparation of TauCl

TauCl was synthesized by mixing equimolar amounts of sodium hypochlorite (Aldrich Chemical, Milwaukee, MI, USA) and taurine (Sigma, St. Louis, MO, USA). TauCl formation was verified by UV absorption (200 to 400 nM) [27]. Endotoxin-free or low-endotoxin grade water and buffers were used. Stock solutions of taurine and TauCl were kept at 4°C and used within 3 days.

### Semi-quantitative RT-PCR

TRIzol^® ^reagent (Invitrogen, **Carlsbad, CA, USA**) was used to extract total RNA from arthritic FLSs (2.5 × 10^5 ^cells/60-mm dish/2 ml serum-free media) that had been starved in serum-free media overnight and treated with IL-1β for 6 hours in the presence or absence of TauCl. Complementary DNA was synthesized from 1 μg total RNA in 20 μl reverse transcription reaction mixture containing 5 mmol/l MgCl_2_, 1× RT buffer, 1 mmol/l dNTP, 1 U/μl RNase inhibitor, 0.25 U/μl AMV reverse transcriptase, and 2.5 μmol/l random 9-mers. For semi-quantitative PCR, aliquots of cDNA were amplified with the primers in a 25 μl PCR mixture containing 1× PCR buffer, 0.625 units of TaKaRa Ex Taq™ HS, and 0.2 μmol/l of specific upstream primers, in accordance with the manufacturer's protocol (TaKaRa Bio, Kyoto, Japan). The PCR conditions for the MMPs were as follows: 30 to 33 cycles at 95°C for 45 s, 55 to 60°C for 45 s, and 72°C for 45 s. PCR products were subjected to electrophoresis in 1.5% agarose gels containing ethidium bromide, and the bands were visualized under UV light. The primers were synthesized by Bioneer Co. Ltd (Seoul, Republic of Korea), and their sequences are listed in Table 1.

**Table 1:** The sequence of PCR primers used in this experiment

Primer name	Primer sequence	Product size
MMP-1 sense	5'-CCT AGC TAC ACC TTC AGT GG-3'	338 bp
MMP-1 antisense	5'-GCC CAG TAC TTA TTC CCT TT-3'	
MMP-13 sense	5'-TTG AGG ATA CAG GCA AGA CT-3'	311 bp
MMP-13 antisense	5'-TGG AAG TAT TAC CCC AAA TG-3'	
MMP-2 sense	5'-ACT TCA GGC TCT TCT CCT TT-3'	288 bp
MMP-2 antisense	5'-TTC AGA CAA CCT GAG TCC TT-3'	
MMP-9 sense	5'-TAC CCT ATG TAC CGC TTC AC-3'	345 bp
MMP-9 antisense	5'-GAA CAA ATA CAG CTG GTT CC-3'	
β-actin sense	5'-TCA TGA GGT AGT CAG TCA GG-3'	305 bp
β-actin antisense	5'-CTT CTA CAA TGA GCT GCG TG-3'	

bp, base pairs; MMP, matrix metalloproteinase.

### Real-time PCR

For real-time quantitative PCR analysis, the reaction was carried out using the LightCycler PCR system (Roche Diagnostics, Meylan, France), with the DNA-binding SYBR Green I dye used to detect the PCR products. A serial dilution was used to generate each standard curve. Each 20 μl reaction mixture contained 1× LightCycler-DNA Master SYBR Green I, a specific primer, along with 2 μl cDNA. After 2 min denaturation at 95°C, the MMPs and β-actin underwent 40 reaction cycles at 95°C for 5 s, 55 to 60°C for 10 s (annealing) and 72°C for 13 s. Product specificity was determined by melting curve analysis, as described in the LightCycler manual. The results are expressed as ratios of MMP transcripts to β-actin transcripts, with the quantity of transcripts in each sample expressed as a copy number. The ratio of MMP/β-actin mRNA was assigned a value of 100%, with the corresponding results calculated as relative percentages.

### Enzyme-linked immunosorbent assay

The levels of MMP-1 and MMP-13 secreted in the culture media by IL-1β stimulated FLSs (5 × 10^5 ^cells/60-mm dish/2-ml serum-free media) in the presence or absence of TauCl were measured by ELISA (R&D Systems, Inc., Minneapolis, MN, USA).

### Western blot analysis

FLSs (5 × 10^5 ^cells) cultured in 60-mm dishes were serum starved overnight and stimulated by IL-1β (10 ng/ml) for 10 or 30 min in the presence or absence of TauCl. The cells were subsequently washed twice in phosphate-buffered saline (PBS) and treated with 50 μl lysis buffer (20 mmol/l Tris-Cl [pH 8.0], 150 mmol/l NaCl, 1 mmol/l EDTA, 1% Triton X-100, 20 μg/ml chymostatin, 2 mmol/l phenylmethylsuphonyl fluoride [PMSF], 10 μmol/l leupeptin, and 1 mmol/l 4-[2-aminoethyl]benzenesulfonyl fluoride). Cells were scraped using a rubber policeman before addition of another 50 μl lysis buffer. The cells were transferred to a microcentrifuge tube, incubated on ice for 30 min with occasional agitation every 5 min and centrifuged for 15 min at 12,000 rpm (16,090 *g*), and the supernatant was then analyzed for protein concentration using the Bio-Rad Protein Assay Kit (Bio-Rad, Hercules, CA, USA). Thirty micrograms of cytoplasmic protein extract were then boiled in 5× Laemmli sample buffer for 5 min. The samples were separated by 12% SDS-PAGE and transferred to a Hybond-ECL membrane (Amersham, Arlington Heights, IL, USA). The membranes were blocked with 6% nonfat milk dissolved in TBST buffer (10 mmol/l Tris-Cl [pH 8.0], 150 mmol/l NaCl, 0.05% Tween 20). The blots were probed with various rabbit polyclonal antibodies for phosphorylated extracellular signal regulated kinase-1/2 (phospho-ERK-1/2), phospharylated p38 (phospho-p38), phospharylated c-jun amino-terminal kinase (phospho-JNK), and inhibitor of nuclear factor-κB (IκB)α (Cell Signaling Technology, Beverly, MA, USA) diluted 1:1000 in Tris-buffered saline for 2 hours and incubated with 1:1000 dilutions of goat anti-rabbit IgG secondary antibody, coupled with horseradish peroxidase. The blots were developed using the ECL method (Amersham). For re-probing, the blots were incubated in the stripping buffer (100 mmol/l 2-mercaptoethanol, 2% SDS, 62.5 mmol/l Tris-HCl [pH 6.7]) at 50°C for 30 min with occasional agitation.

### Preparation of nuclear extracts

FLSs (2 × 10^6 ^cells) were seeded in 100-mm dishes and cultured for 2 days. The cells were kept in serum-free medium overnight and pretreated with TauCl 30 min before IL-1β (10 ng/ml) stimulation for 90 min. The cells were then washed with cold PBS, and nuclear extracts were prepared by cell lysis followed by nuclear lysis. In brief, cells were suspended in 400 μl of buffer A (10 mmol/l HEPES [pH 7.9], 1.5 mmol/l MgCl_2_, 10 mmol/l KCl, 0.5 mmol/l DTT, 1 μmol/l leupeptin and 0.2 mmol/l PMSF) and vortexed for 15 s. After incubation for 20 min at 4°C, the lysates were centrifuged at 10,000 *g *for 6 min. The unclear pellet was re-suspended in buffer B (20 mmol/l HEPES [pH 7.9], 25% glycerol, 420 mmol/l NaCl, 1.5 mmol/l MgCl_2_, 0.2 mmol/l EDTA, 0.5 mmol/l DTT, 1 μmol/l leupeptin and 0.2 mmol/l PMSF), incubated on ice for 40 min and centrifuged at 10,000 *g *for 20 min. Protein concentrations were determined using the Bradford method (Bio-Rad).

### Electrophoretic mobility shift assay

The protein-DNA binding activity in nuclear factor-κB (NF-κB) was determined using electrophoretic mobility shift assay (EMSA). In brief, 10 μg nuclear protein was incubated with 0.25 μg of poly(dI-dC) (Amersham) and ^32^P-labelled DNA probe (5,000 counts per minute) in binding buffer (10 mmol/l Tris-HCl [pH 7.5], 50 mmol/l NaCl, 1 mmol/l MgCl_2_, 0.5 mmol/l EDTA, 5% glycerol and 0.5 mmol/l DTT) for 30 min at 30°C. The protein-DNA complexes were then analyzed on 5% native polyacrylamide gels. For the supershift experiment, antibodies were included in the above reaction mixture and incubated at 4°C for 3 hours before the addition of the ^32^P-labelled DNA probe. The oligonucleotide sequences used to detect NF-κB activity were as follows: 5'-AGT TGA GGG GAC TTT CCC AGG-3' (sense) and 5'-GCC TGG GAA AGT CCC CTC AAC T-3' (antisense).

### Immunofluorescence staining

FLSs were cultured at 4 × 10^4 ^cells/well in four-well Lab-Tek chamber slides (Falcon; Becton Dickinson Labware, Oxnard, CA, USA) in order to visualize the translocation of NF-κB to the nucleus under IL-1β stimulation. After serum starvation overnight, the cells were stimulated with IL-1β at 10 ng/ml for 90 min, washed with cold PBS, and fixed with 4% paraformaldehyde in PBS for 20 min. Cells were permeabilized with PBS and 0.5% Triton X-100 in PBS for 10 min, and were then incubated for 30 min with the blocking buffer, 5% goat serum, in order to prevent nonspecific binding. The cells were incubated with 5 μg/ml rabbit polyclonal anti-NF-κB p65 antibody (Santa Cruz Biotechnology) overnight, followed by incubation with 20 μg/ml cyan3-conjugated goat anti-rabbit antibody for 60 min at room temperature. After washing, the cells were counterstained with 0.1 mg/ml DAPI for 30 min at room temperature. The coverslips were fixed with mounting media (DakoCytomation, Carpinteria, CA, USA), and the slides were visualized using confocal microscopy (Carl Zeiss, Oberkochen, Germany).

### *In vitro *cytotoxicity

TauCl cytotoxicity was assessed by a colorimetric assay using 3-(4,5-dimethylthiazol-2-yl)-2,5-diphenyltetrazolium bromide (MTT). In brief, FLSs (2.5 × 10^5 ^cells/2 ml) were seeded into six-well plates. After overnight incubation at 37°C, the medium was replaced with serum-free medium and treated with TauCl 30 min before stimulation with IL-1β (10 ng/ml). Cells were subsequently cultured for 24 hours, and MTT solution (5 mg/ml) was added to each well at a final concentration of 0.5 mg/ml. The plates were incubated for 4 hours at 37°C, and formazan crystals were dissolved by the addition of 1 ml isopropanol containing 0.04 M HCl. Finally, absorbance was measured at 595 nm.

### Statistical analysis

All experiments were repeated three times, and the results are expressed as the mean ± standard deviation. Statistical evaluation was performed by means of a paired Student's *t*-test. Differences were considered statistically significant at *P *< 0.05.

## Results

### TauCl differentially inhibits the expression of MMP-1 and MMP-13

**Figure 1:**
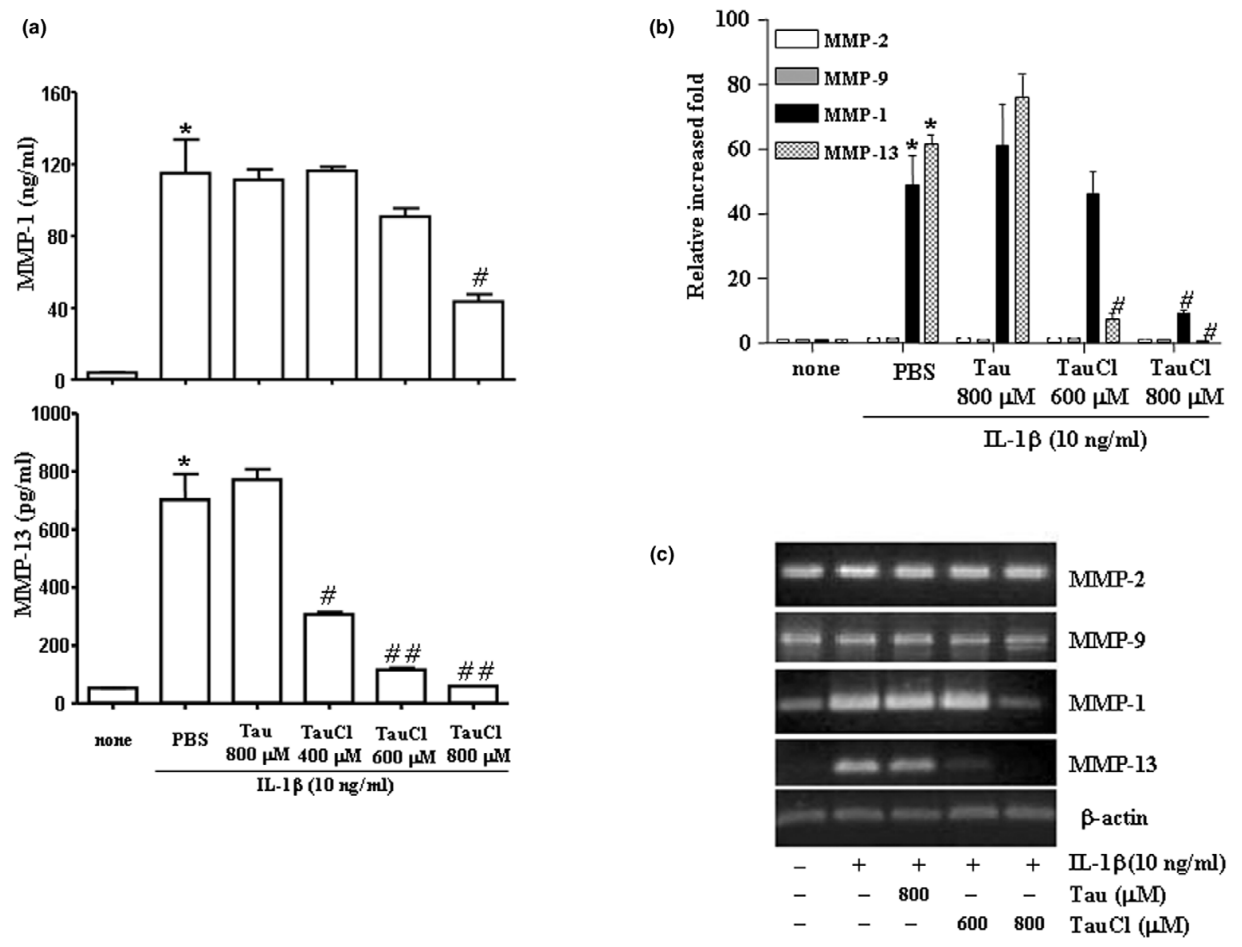
TauCl differentially inhibits the expression of MMPs in IL-β-stimulated RA FLSs. The expressions of the collagenases (matrix metalloproteinase [MMP]-1 and MMP-13) and the gelatinases (MMP-2 and MMP-9) were determined by **(a) **ELISA analysis, **(b) **real time PCR and **(c) **semi-quantitative RNA analysis. Synovial cells (5 × 10^5 ^cells/60 mm dish/2 ml serum-free media) were treated with taurine chloramine (TauCl) 30 min before 24 hours of IL-1β (10 ng/ml) stimulation for MMP protein analysis by ELISA. Cells (2.5 × 10^5 ^cells/60 mm dish/2 ml serum-free media) were treated with TauCl 30 min before 6 hours of stimulation with IL-1β (10 ng/ml) for RNA level analysis. IL-1β stimulated the expression of the MMP-1 and MMP-13 genes, but it did not affect the expression of MMP-2 or MMP-9. TauCl differentially inhibited the expressions of MMP-1 and MMP-13. Experiments were performed in duplicate with cells from three patients. Values are expressed as means ± standard deviation. *P < 0.01 versus control group (no IL-1β); ^#^*P *< 0.05 and ^##^*P *< 0.01 versus IL-1β treatment group without TauCl. FLS, fibroblast-like synoviocyte; PBS, phosphate-buffered saline; RA, rheumatoid arthritis.

The stimulation of arthritic FLSs using IL-1β greatly upregulated the expressions of the MMP-1 and MMP-13 collagenases, as determined by ELISA (Figure 1a), real time PCR (Figure 1b) and semi-quantitative RNA analysis (Figure 1c). However, the expressions of the gelatinases MMP-2 and MMP-9 remained unchanged (Figure 1). MMP-2 and MMP-9 remained unchanged at the mRNA level, even after 24 hours of stimulation, which indicates that IL-1β did not stimulate MMP-2 and MMP-9 (data not shown). Consistent with the mRNA levels of MMP-1 and MMP-13, ELISA analyses of culture supernatants at 24 hours revealed that IL-1β upregulated the expressions of MMP-1 and MMP-13 at the protein level by about 30-fold and 15-fold, respectively (Figure 1a). The protein levels of the MMP-2 remained unchanged, whereas the protein levels of the MMP-9 genes were below the ELISA detection limit (data not shown).

To identify whether TauCl inhibits the expression of MMPs, FLSs were treated with TauCl 30 min before 24 hours or 6 hours of IL-1β stimulation for protein analysis and RNA analysis, respectively. Treatment with TauCl at concentrations of 400 and 600 μmol/l differentially inhibited the expressions of MMP-1 and MMP-13. The expression of MMP-1 remained unchanged at TauCl 400 and 600 μmol/l, whereas MMP-13 levels were reduced to about 50% or 20% of that observed in the IL-1β treated group (with no TauCl treatment), respectively (Figure 1a). However, at a TauCl concentration of 800 μmol/l, the protein expressions of MMP-1 and MMP-13 diminished to 50% and 10%, respectively.

Consistent with the effects of TauCl on the protein levels of MMP-1 and MMP-13, RNA analyses revealed that the levels of MMP-13 were more sensitive to TauCl at a concentration of 600 μmol/l than were the levels of MMP-1. At a TauCl concentration of 800 μmol/l, the transcriptional expressions of the MMP-1 and MMP-13 genes diminished to 20% and 5%, respectively (Figure 1b,c).

### IL-1β stimulates the signalling pathways of both MAPK and IκB kinase

**Figure 2:**
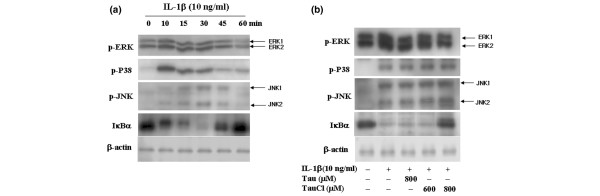
TauCl primarily inhibited the degradation of IκB. **(a) **Synovial cells (5 × 10^5 ^cells/60 mm dish/2 ml serum-free media) were treated with IL-1β (10 ng/ml). Shown are time courses of the signalling pathways activated during IL-1β stimulation. **(b) **Synovial cells (5 × 10^5 ^cells/60 mm dish/2 ml serum-free media) were treated with taurine chloramine (TauCl) 30 min before 10 or 30 min of IL-1β (10 ng/ml) stimulation for Western blot analysis. A TauCl concentration of 800 μmol/l significantly inhibited the inhibitor of nuclear factor-κB (IκB)/nuclear factor-κB (NF-κB) signalling pathway by inhibiting the degradation of IκBα. The mitogen-activated protein kinase (MAPK) signalling pathway, including extracellular signal-regulated kinase (ERK)-1/2, p38 and c-jun amino-terminal kinase (JNK), was unaffected. Three independent experiments were performed with cells from two patients. p, phosphorylated.

IL-1β stimulates the signal transduction pathways of both mitogen-activated protein kinase (MAPK) and IκB kinase in chondrocytes and astrocytes [28,29]. To identify the pathways that are involved in enhancing MMP-1 and MMP-13 expression under IL-1β stimulation, the levels of phospho-ERK-1/2, phospho-JNK, phospho-p38MAPK and IκBα were measured according to the duration of stimulation. IL-1β treatment led to remarkable increases in the phosphorylation of ERK-1/2 and p38MAPK by 10 min, and the increased levels were maintained for 45 min. The level of phospho-JNK peaked at 30 min. IκBα was completely degraded at 30 min but recovered by 60 min, which indicates that IκBα was fully phosphorylated within 30 min of activation of IL-1β (Figure 2a).

### TauCl primarily inhibits the IκBα pathway

**Figure 3:**
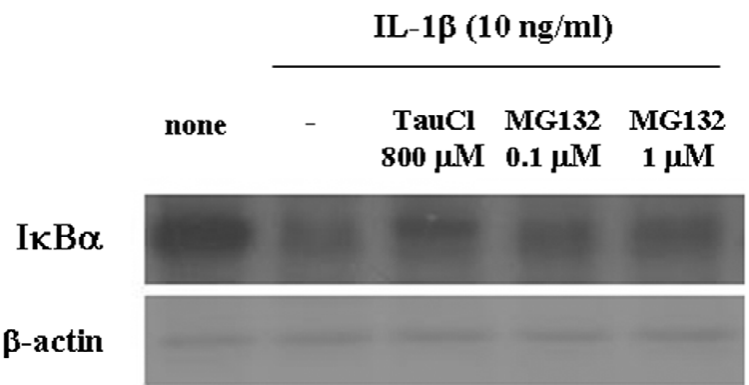
TauCl inhibited IκBα degradation as potently as did a NF-κB inhibitor (MG132). Synovial cells (5 × 10^5 ^cells/60 mm dish/2 ml serum-free media) were treated with taurine chloramine (TauCl) or MG132 30 min before IL-1β (10 ng/ml) stimulation for 30 min. At a concentration of 800 μmol/l, TauCl inhibited the degradation of inhibitor of nuclear factor-κB (IκB)α just as potently as did 1 μmol/l MG132. Three independent experiments were performed with cells from two patients. NF-κB, nuclear factor-κB.

We investigated the level of MAPK phosphorylation in an effort to clarify the inhibitory mechanism of TauCl on MMPs. As shown in Figure 2b, TauCl did not significantly inhibit the phosphorylation of ERK-1/2, p38, or JNK, even when the highest test concentration of 800 μmol/l was used. At 800 μmol/l, TauCl strongly blocked the IκB degradation that normally occurs upon IL-1β stimulation, which suggests that TauCl prevents NF-κB from migrating to the nucleus by inhibiting the degradation of IκB. The effectiveness of TauCl as an inhibitor of IκB was investigated by comparing it with MG132, which is an NF-κB inhibitor that slows IκB degradation by deactivating the proteasome [30]. TauCl inhibited IκB degradation as potently as MG132, in this instance; however, the concentration of TauCl employed was greater than that of MG132 (Figure 3).

### TauCl blocks NF-κB nuclear translocation through the inhibition of IκB degradation

**Figure 4:**
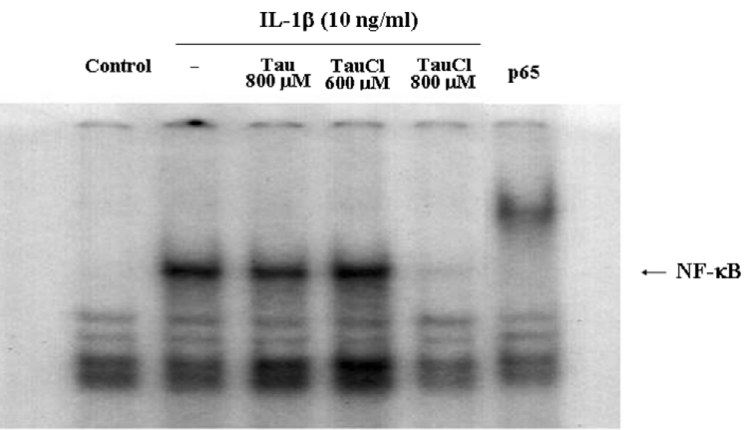
TauCl inhibited NF-κB binding activity. Synovial cells (2 × 10^6 ^cells/100-mm dish/5-ml serum-free media) were pretreated with taurine chloramine (TauCl) or taurine (Tau) 30 min prior to IL-1β stimulation for 90 min. Nuclear extracts were prepared for electrophoretic mobility shift assay (EMSA). IL-1β stimulation increased nuclear levels of nuclear factor-κB (NF-κB). At a concentration of 800 μmol/l, TauCl completely inhibited NF-κB binding. Antibodies against the p65 subunit of NF-κB induced a gel shift in the NF-κB band. Three independent experiments were performed with cells from two patients.

**Figure 5:**
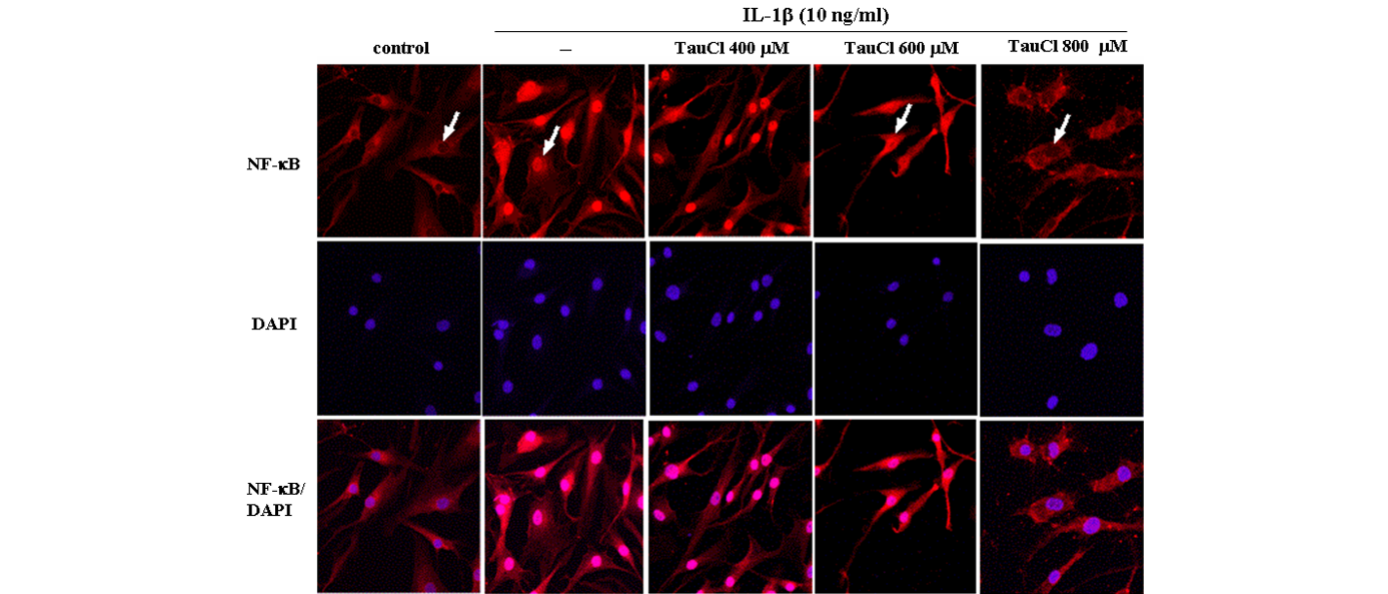
TauCl inhibited the migration of NF-κB into the nucleus. To visualize the translocation of nuclear factor-κB (NF-κB), synovial cells (4 × 10^4 ^cells/well in four-well Lab-Tek chamber slides) were cultured. After serum starvation overnight, the cells were treated with taurine chloramine (TauCl) 30 min before stimulation with IL-1β (10 ng/ml) for 90 min. IL-1β stimulation induced the migration of NF-κB from the cytoplasm into the nucleus (second column), whereas NF-κB was found only in the cytoplasm of nonstimulated cells (first column). At a concentration of 800 μmol/l, TauCl completely inhibited the migration of NF-κB into the nucleus (fifth column). All pictures were taken at a magnification of 200×. Three independent experiments were performed in duplicate with cells from two patients.

To further demonstrate that the effects of IκB degradation extended to the transnuclear migration of NF-κB, levels of NF-κB in the nucleus were assessed using EMSA (Figure 4) and immunohistochemistry (Figure 5). As shown in Figure 4, at a concentration of 800 μmol/l, TauCl completely blocked the nuclear binding of NF-κB; however, at 600 αmol/l, TauCl did not block binding activity. These results were confirmed by confocal microscopy. After 90 min of IL-1β stimulation, the majority of cytoplasmic NF-κB migrated into the nucleus, as indicated by strong nuclear NF-κB staining following stimulation and strong cytoplasmic staining before stimulation (Figure 5). Confirming previous findings, the migration of NF-κB into the nucleus was not inhibited at TauCl concentrations of up to 600 μmol/l. However, at a concentration of 800 μmol/l, TauCl blocked the transnuclear migration of NF-κB.

## Discussion

Because IL-1β is believed to play a major role in synovial inflammation, RA FLSs stimulated with IL-1β *in vitro *have been used to mimic the synovial proliferation that occurs in RA patients suffering from inflammation [31]. IL-1β is also known to stimulate many proinflammatory mediators in a variety of cell types [32]. In addition, IL-1β is a potent inducer of metalloproteinase production by FLSs; however, little investigation has been conducted to determine its effects on the gelatinases (MMP-2 and MMP-9) [33]. In the present study, we found that IL-1β strongly stimulated the expression of collagenases (MMP-1 and MMP-13). Gelatinase expression was weakly activated by IL-1β stimulation. However, IL-1β is known to induce high levels of gelatinase expression in other cell types [34-36].

IL-1β activates different signalling pathways in different cell types. Thus, we investigated signalling pathways in IL-1β stimulated RA FLSs [37]. IL-1β stimulated the pathways of both MAPK (ERK, p38 and JNK) and IκB kinase within 30 min, with pathway activation subsiding to the basal levels of nonstimulated cells by 60 min. The activation of these pathways led to the activation of a number of transcriptional factors that enhance the expression of various proinflammatory mediators. Among these factors, NF-κB is a key regulator of inflammatory gene transcription, and it is known to be activated in RA synovia and chondrocytes [38].

TauCl differentially inhibited the expression of MMPs in IL-1β stimulated RA FLSs. The expression of MMP-13 was significantly inhibited at concentrations of 400 to 600 μmol/l TauCl, whereas the expression of MMP-1 was not significantly inhibited at this concentration. To clarify the inhibitory mechanism of TauCl on MMPs, the levels of both MAPK phosphorylation and IκB degradation were investigated in IL-1β stimulated RA FLSs. TauCl did not significantly inhibit the phosphorylation of ERK-1/2, p38, or JNK, even at 800 μmol/l, whereas IκB degradation was significantly inhibited at 800 μmol/l. These findings indicate that the inhibition of the IκB signalling pathways by TauCl was primarily dependent on the inhibition of IκB degradation. This finding is consistent with previous reports showing that TauCl modifies the backbone of IκB through amino acid oxidation of IκB, thus allowing IκB to become resistant to degradation [39,40]. Confocal microscopic examination of the NF-κB immunostaining results indicated that a TauCl concentration of 800 μmol/l was required to inhibit IκB degradation completely. Partial inhibition of IκB degradation was seen at a TauCl concentration of 600 μmol/l, as reflected by NF-κB immunostaining in both the cytoplasm and the nucleus. This may indicate that signalling pathways other than the MAPK and IκB pathways are involved in the stimulation of MMP-1 and MMP-13. In support of this idea, protein kinase Cδ is known to play a key role in the stimulation of MMP-13 via crosstalk with MAPKs in basic fibroblast growth factor stimulated human adult articular chondrocytes [41]. At concentrations lower than 800 μmol/l, TauCl may inhibit or block minor pathways that are involved in the upregulation of MMP-1 and MMP-13. At a critical concentration (600 to 800 μmol/l), IκBα degradation is completely inhibited, thereby preventing the migration of NF-κB into the nucleus.

**Figure 6:**
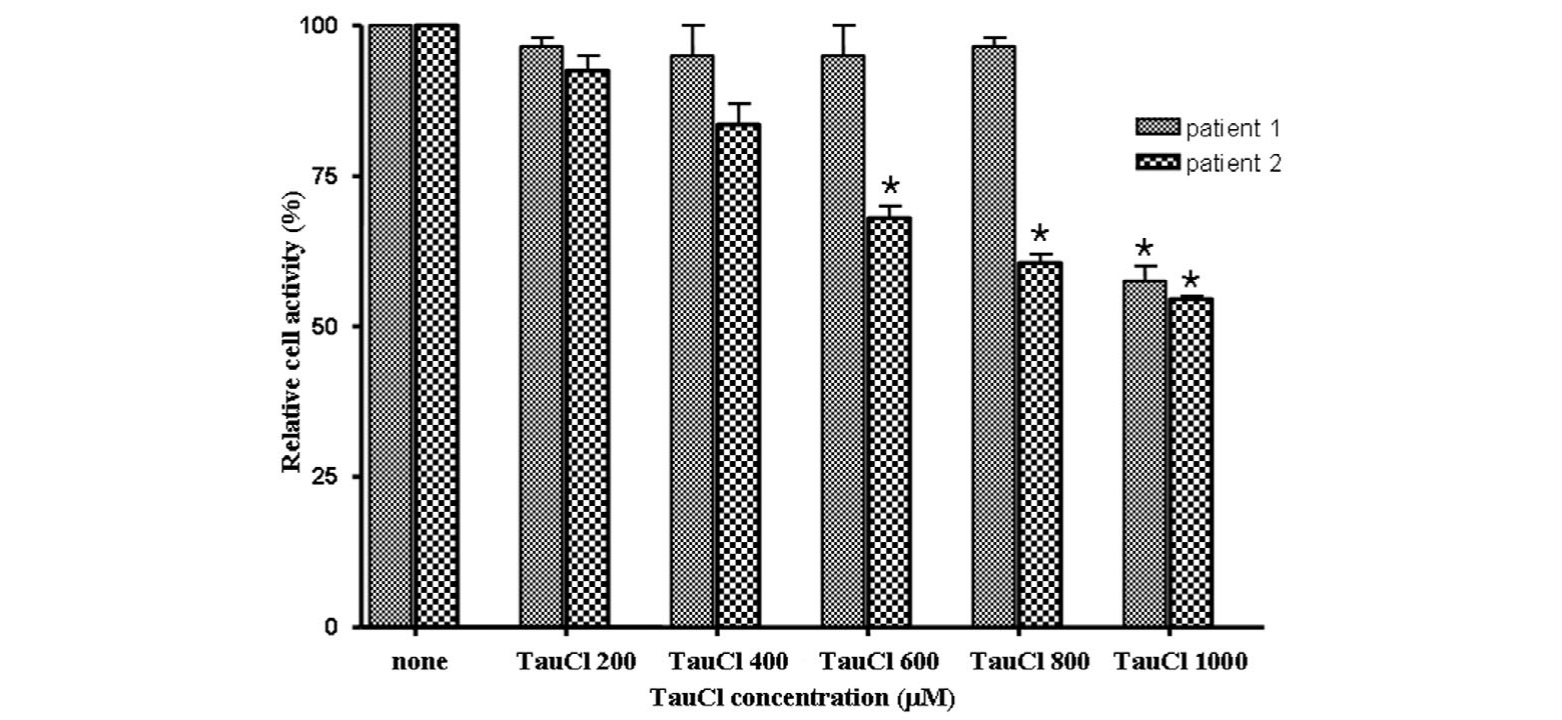
Effect of TauCl on the viability of RA FLSs. Rheumatoid arthritis (RA) fibroblast-like synoviocytes (FLSs) from two RA patients were treated with taurine chloramine (TauCl) 30 min before the stimulation with IL-1β (10 ng/ml), and were incubated for 24 hours (as described in Materials and methods). Cell activity was then determined by 3-(4,5-dimethylthiazol-2-yl)-2,5-diphenyltetrazolium bromide (MTT) assay, and is expressed as the mean ± standard deviation of three separate experiments. Three independent experiments were performed with cells from two patients. *P < 0.05 versus untreated control.

TauCl is less toxic than its precursor HOCl/OCl^-^, but cytotoxic effects of TauCl at high concentrations have been reported. Its toxicity appears to differ between cell types [42]. Kontny and coworkers [43] reported that TauCl caused progressive necrosis of RA FLSs at concentrations of 500 μmol/l or greater. In our study, the RA FLSs used in the experiments were not significantly affected by a TauCl concentration of 800 μmol/l for 24 hours, even though cytotoxicity was detected in RA FLSs from some patients (Figure 6). TauCl toxicity appeared to vary between individual RA patients. In addition, different cell passages might have contributed to the variance in sensitivity to TauCl, because RA FLSs exhibit different characteristics according to passage [44,45]. Although it remains uncertain whether the TauCl concentration used in this experiment can be a physiologic concentration, TauCl may remain at a high concentration in extracellular fluids because the intracellular and extracellular concentrations of taurine in mammalian tissues are 10 to 70 mmol/l and 20 to 100 μmol/l, respectively [46].

The differential effects of TauCl on the expressions of MMP-1 and MMP-13 may also be related to other transcription factors that are differentially involved in the activations of MMP-1 and MMP-13. For example, Runxa2 was found to stimulate strongly the transcriptional activation of MMP-13, but it had no effect on MMP-1 expression in human chondrosarcoma cells [47]. In addition, many transcriptional binding sites, such as activator protein-1 and Ets/polymavirus enhancer 3 (OSE-2), have been identified in the human MMP-13 proximal promoter [48-50]. An AG-rich element regulatory site was recently found in the human MMP-13 proximal promoter [51]. This and other transcription factors may contribute to the increased expression of MMP-13 in IL-1β stimulated FLSs. The interaction of TauCl with these as yet unidentified factors remains unknown. Furthermore, these transcription factors may function at a TauCl concentration that inhibits the degradation of IκB.

The degree of the inhibitory effect of TauCl was compared with that of an NF-κB inhibitor, namely MG132. At a concentration of 800 μmol/l, the inhibitory effect of TauCl on IκB degradation was as potent as that of 1 μmol/l MG132. Because MMP-13 exhibits the greatest activity toward the degradation of type II collagen, a major component of the cartilage extracellular matrix, the control of MMP-13 expression is crucial when attempting to delay the degradation of cartilage [26]. At lower concentrations of TauCl, inhibition of MMP-13 expression would be a potentially effective strategy to control the destruction of joint cartilage in RA and osteoarthritis. Above all, TauCl may be produced as a part of the homeostatic response to infection and inflammation, thus playing a critical role in limiting the duration and intensity of immune inflammation [52]. In support of this hypothesis, synovial fluid neutrophils of RA patients exhibit impaired generation of TauCl [53].

In summary, TauCl differentially inhibited the increased expression levels of MMP-1 and MMP-13 in IL-1β stimulated RA FLSs. It inhibited the expression of MMP-1 primarily through inhibition of IκB degradation, although it did not appear to inhibit the expression of MMP-13 through inhibition of the IκB signalling pathway.

## Conclusion

Given that MMP-13, which is inhibited by TauCl, is remarkably active against collagen type II, and that synovial fluid neutrophils of RA patients exhibit impaired generation of TauCl, the involvement of TauCl in destruction of joint cartilage should receive greater focus. This may yield insights into the molecular mechanisms of joint destruction in RA.

## Abbreviations

ELISA = enzyme-linked immunosorbent assay; EMSA = electrophoretic mobility shift assay; ERK = extracellular signal-regulated kinase; FLS = fibroblast-like synoviocyte; HOCl = hypochlorous acid; IκB = inhibitor of nuclear factor-κB; IL = interleukin; JNK = c-jun amino-terminal kinase; MAPK = mitogen-activated protein kinase; MMP = matrix metalloproteinase; MTT = 3-(4,5-dimethylthiazol-2-yl)-2,5-diphenyltetrazolium bromide; NF-κB = nuclear factor-κB; PBS = phosphate-buffered saline; PCR = polymerase chain reaction; PMSF = phenylmethylsuphonyl fluoride; RA = rheumatoid arthritis; TauCl = taurine chloramines.

## Competing interests

The authors declare that they have no competing interests.

## Authors' contributions

KSK participated in the data analysis and the design of the study, and drafted the manuscript. EKP, SMJ, H-SJ and JSB performed the experiments. CK supplied TauCl, performed EMSA and helped to edit the manuscript. Y-AL, S-JH, S-HL and H-IY provided clinical perspectives regarding the relation of TauCl with RA. MCY provided the synovium from patients and participated in the design of the study. All authors read and approved the final manuscript.
